# Accuracy of Acetabular Cup Position Using CT Navigation During Total Hip Arthroplasty in Patients With Developmental Dysplasia of the Hip

**DOI:** 10.7759/cureus.87865

**Published:** 2025-07-13

**Authors:** Takuya Nakai, Shigeo Fukunishi

**Affiliations:** 1 Orthopedic Surgery, Nishinomiya Kaisei Hospital, Hyogo, JPN

**Keywords:** ct-based navigation systems, cup position, double floor, three-dimensional accuracy, total hip arthroplasty

## Abstract

Background

CT-based navigation systems for total hip arthroplasty (THA) have been used to prevent complications in cup placement. It has been reported that the accuracy of cup alignment, known as inclination and anteversion, has improved with the use of a navigation system. However, while cup placement height and medialization are important, there are few reports on the accuracy of cup positioning.

Methods

CT-based navigation was used for cup placement, and the discrepancy between preoperative planning and postoperative CT evaluation was examined in three dimensions. It was also investigated whether the degree of developmental dysplasia of the hip (DDH) (Crowe classification and double floor) made a difference in the accuracy of cup placement.

Results

The absolute discrepancies between preoperative plans and postoperative measurements were 1.7 (range 0-8) mm in the transverse axis, 2.3 (range 0-12) mm in the sagittal axis, and 2.2 (range 0-11) mm in the longitudinal axis. No significant variation was found in any of the three-dimensional measurements taken for all Crowe classifications, with or without the presence of an acetabular “double floor”. However, the double-floor subgroup showed a trend toward greater discrepancy in all directions.

Conclusions

CT-based navigation systems can position the cup with high three-dimensional accuracy in primary THA without affecting acetabular deformity.

## Introduction

Total hip arthroplasty (THA) has been reported to have a high survival rate and excellent patient satisfaction [1,2]. Acetabular cup placement is critical to clinical outcome and long-term survival of THA. Furthermore, acetabular cup malalignment has been attributed to an increased rate of impingement, dislocation, cup migration, and polyethylene wear [3]. Assisted surgical tools, such as mechanical guides and both CT-free and CT-based navigation systems, have been used to improve accuracy as well as prevent complications of cup placement [4-7]. We have previously reported on the accuracy of cup placement using CT-based navigation (radiographic cup inclination: 39.8 ± 2.7° for a target of 40°, radiographic cup anteversion: 13.7° ± 3.5° for a target of 15°) [8]. Recently, robotic arm-assisted systems have been developed, which have a higher accuracy for cup alignment than navigation systems. Okazaki et al. compared the accuracy of postoperative cup alignment between robotic THA and CT-navigated THA, reporting that the absolute discrepancy was 1.4° ± 1.2° vs. 2.7° ± 2.2° in inclination and 1.5° ± 1.3° vs. 2.2° ± 1.7° in anteversion, showing that the accuracy of robotic THA was higher than that of CT-navigated THA [9].

However, cup positioning is equally as important as alignment for successful THA. Therefore, the height of cup placement and the degree of medialization must be determined to ensure adequate cup coverage of the host bone [10-12]. The height at which the cup is placed is related to the adjustment of leg length, while medialization is related to obtaining proper offset and tension in the abductor muscles [13-15]. In addition, attention has been paid to the impingement of the iliopsoas muscle due to the anterior overhang of the cup, and, in recent years, the position of the cup in the coronal plane has also been considered important [16-18]. Preoperative planning is left to the surgeon's discretion as to whether to place the cup in the original acetabulum and to what height or degree of medialization to allow. It is necessary to conduct a three-dimensional preoperative plan for the appropriate cup, which can then be reproduced intraoperatively. In particular, in preoperative planning for developmental dysplasia of the hip (DDH) patients, various factors are involved in determining the appropriate positioning of the cup [19]. Furthermore, the shallow acetabular cavity, acetabular wall defects, double floor, and contracted soft tissues make it difficult for surgeons to identify the original acetabulum and properly position the cup during surgery [20,21].

The use of a CT-based navigation system during preoperative planning allows intraoperative reproducibility of the longitudinal, transverse, and sagittal positions of the cup. However, there are few reports on the accuracy of cup position. In this study, cup placement was performed using CT-based navigation, and the accuracy was verified by comparison with the preoperative planning. Crowe et al. classified the degree of superior subluxation of the femoral head in DDH patients from I to IV, and this classification is widely used to indicate the degree of DDH [22]. Furthermore, a double floor is recognized as an X-ray finding that indicates lateral subluxation of the femoral head from the original acetabulum to the secondary acetabulum. This study examined the effect of the degree of acetabular deformity associated with DDH on the accuracy of cup placement, with and without a double floor, using the Crowe classification.

This article was previously presented as a meeting abstract at the Japanese Orthopaedic Association Annual Meeting on May 25, 2024.

## Materials and methods

Study design and populations

This study is a retrospective, non-randomized observational study and was approved by the institutional review board of Nishinomiya Kaisei Hospital, Hyogo, Japan. In compliance with the regulations of a retrospective review, we made the research materials available by posting the relevant documents on the institution’s notice board and did not request written consent from each participant.

We retrospectively reviewed 175 hips from 162 patients who underwent primary THA with an uncemented cup between April 2020 and February 2022. Two patients with problems related to the use of intraoperative navigation were excluded. Ultimately, 173 hips from 160 patients were included. Of them, there were: 145 hips with osteoarthritis (OA), 13 hips with idiopathic osteonecrosis (ION), four hips with subchondral insufficiency fracture (SIF), seven hips with femoral neck fracture (FNF), three hips with rheumatoid arthritis (RA), and one hip with rapidly destructive coxarthropathy (RDC).

Preoperative planning

**Figure 1:**
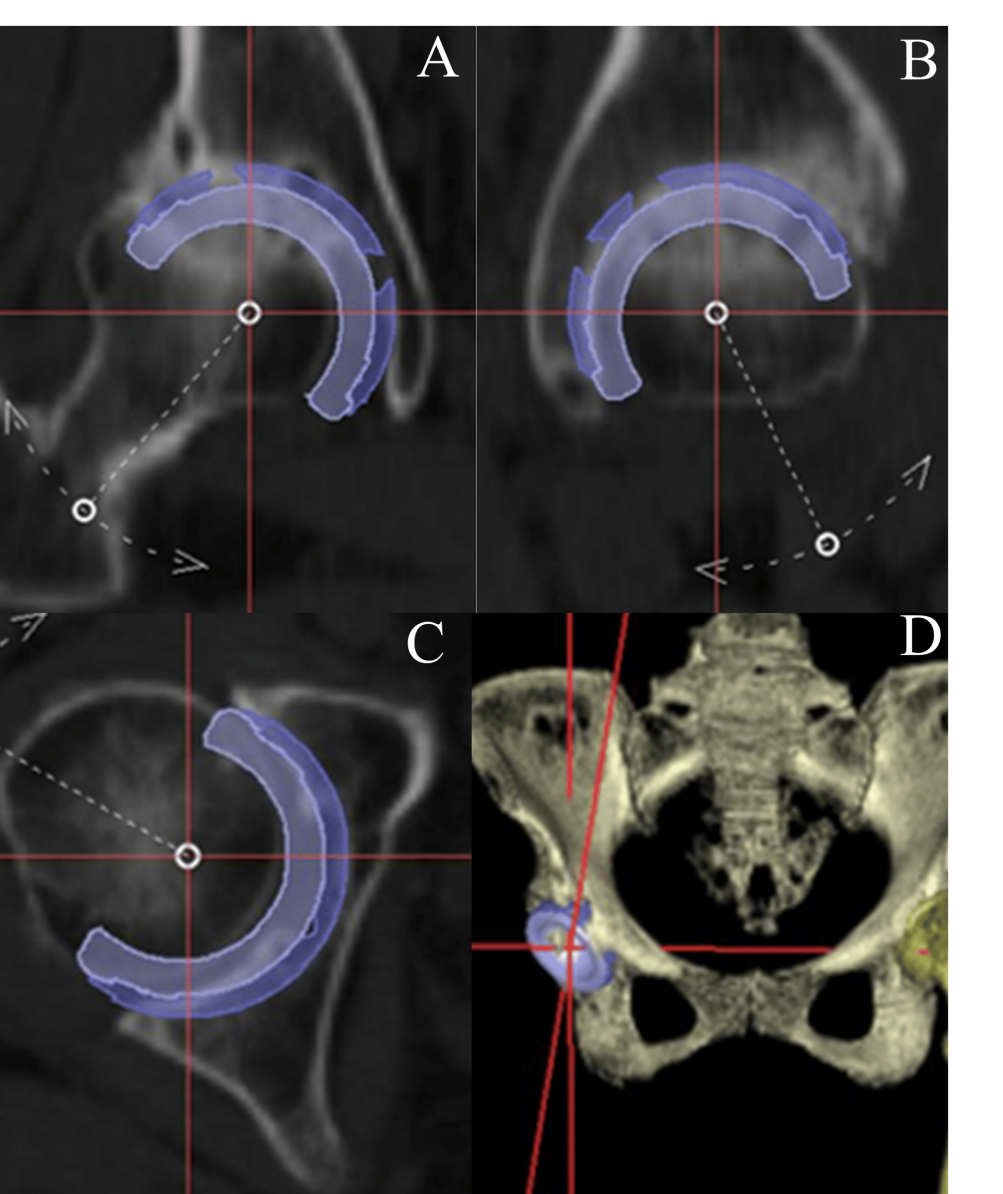
Extraction of coordinates for three-dimensional positioning of cups in preoperative planning. A: Longitudinal slice view B: Sagittal slice view C: Transverse slice view D: Three-dimensional CT

All patients underwent preoperative CT imaging (Aquilion lightning, Canon Medical Systems Corporation, Japan) from the pelvis to the posterior femoral condyle knee joint. Preoperative planning was conducted in the CT-based navigation workstation (CT-based Hip Navigation version 1.1, Stryker Navigation, Germany), and the data were transferred to the workstation in order to calculate the required size, alignment, and position of the cup (Figure1).

The functional pelvic plane (FPP) was utilized as the reference plane for preoperative planning and navigation surgery. The cup position was adjusted to the level of the true acetabulum using a three-dimensional template. Our preoperative planning approach for cup placement is as follows: First, we aim to place the original acetabulum. If the center-edge (CE) angle of the cup cannot be secured at 10°, the cup is medialized to the extent that it does not perforate the medial wall of the acetabulum. Second, if the CE angle of the cup cannot be secured at 10° when medialized, high hip placement up to 10 mm from the tear drop line is permitted. If the cup CE angle still cannot be secured at 10°, an acetabular reconstruction plate and cement cup are selected. Cup size is determined by calculating the cup placement position using the method described above and selecting the cup with the highest cup-to-acetabular contact area ratio in the CT cross section without resulting in anterior or posterior overhang.

Surgical procedure

**Figure 2:**
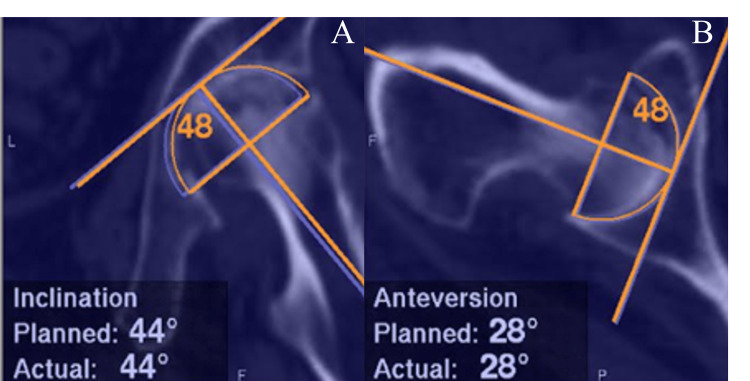
Intraoperative navigation monitor screens A: Anterior-posterior view, the numbers indicate the preoperative planning inclination and the intraoperative reamer inclusion. B: Lateral view, the numbers indicate the preoperative planning anteversion and the intraoperative reamer anteversion.

All surgeries were performed by a single surgeon with extensive experience in THA using the same technique, either the modified Watson-Jones approach or modified Hardinge approach, in all cases from the lateral decubitus position. CT-navigated THA involved the use of landmark and surface matching techniques for pelvic registration, with an acceptable level of accuracy within 1 mm. This was followed by reaming of the acetabulum and placement of the cup with the aid of navigation (Figure 2).

Six points were registered for landmark matching: the superior rim of the acetabulum, the anterior rim of the acetabulum, the posterior rim of the acetabulum, the acetabular fossa, the lunate surface, and the anterior superior iliac spine (ASIS). For surface matching, 30-40 points on the iliac surface around the acetabulum were noted. The acetabular cup was placed according to the preoperative planning and positioned using navigation. A cementless cup (Trident Acetabular Shell, Stryker Orthopedics, USA) was implanted in all cases.

Postoperative evaluations

**Figure 3:**
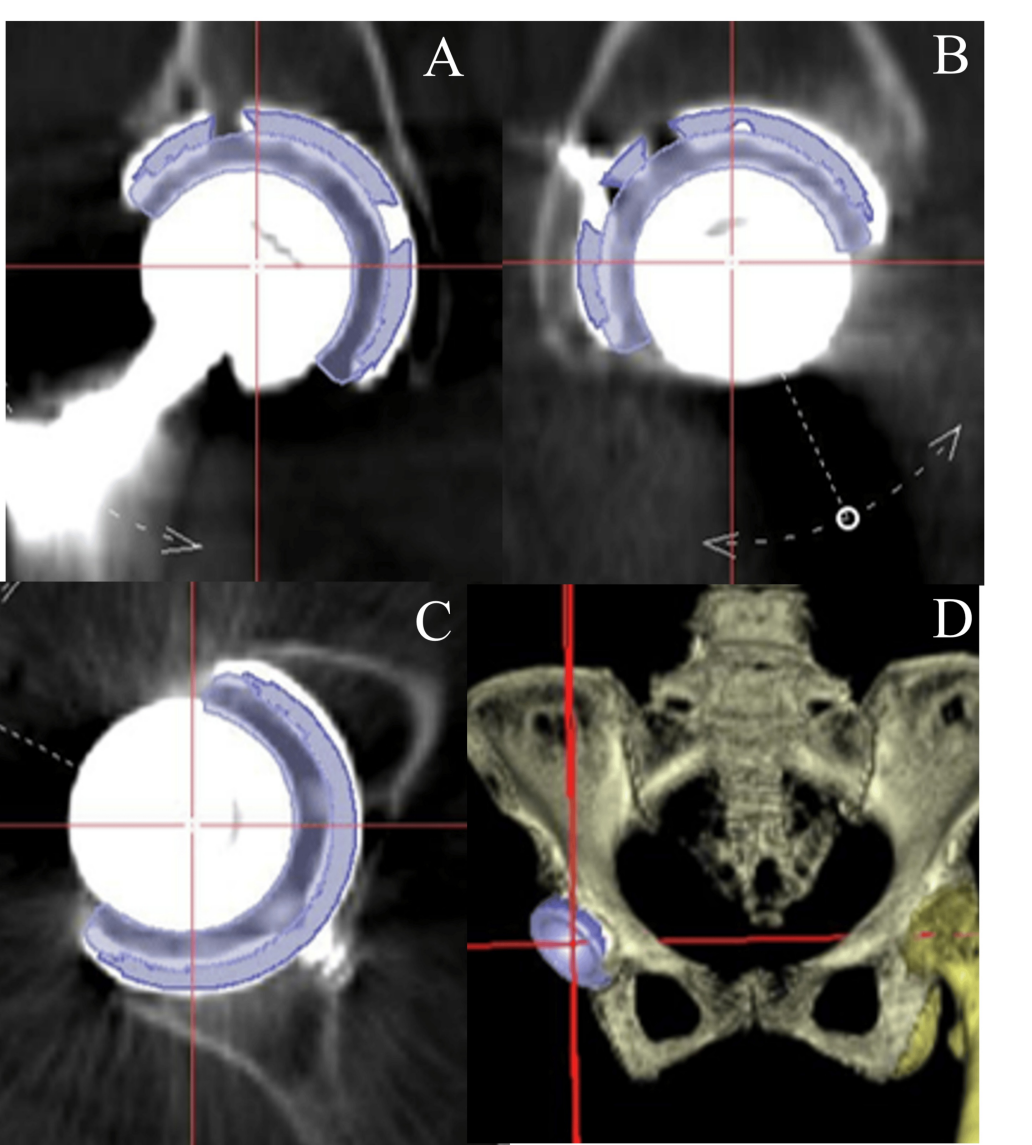
Extraction of coordinates for three-dimensional positioning of cups after surgery in same case with Figure 1 A: Longitudinal slice view B: Sagittal slice view C: Transverse slice view D: Three-dimensional CT

The method for measuring cup position error was based on the research of Tsutsui et al., who demonstrated the effectiveness of cup position accuracy in navigation systems [23]. A CT scan was conducted approximately one week after surgery and the postoperative CT data were transferred to the workstation of the navigation system. A reference plane based on FPP was created from eight reference points on the pelvis; the bilateral ASISs, the bilateral pubic tubercles, the most distal points of the bilateral ischia, the mid-pubic symphysis, and the sacral midplane. Preoperative planning and postoperative cup position were evaluated using a three-dimensional template in the navigation system. To assess postoperative cup position, a full-size cup template was selected, and the cup center, cup anteversion, and cup inclination were superimposed on the implant image in the workstation to quantify the postoperative cup position (Figure 3).

**Figure 4:**
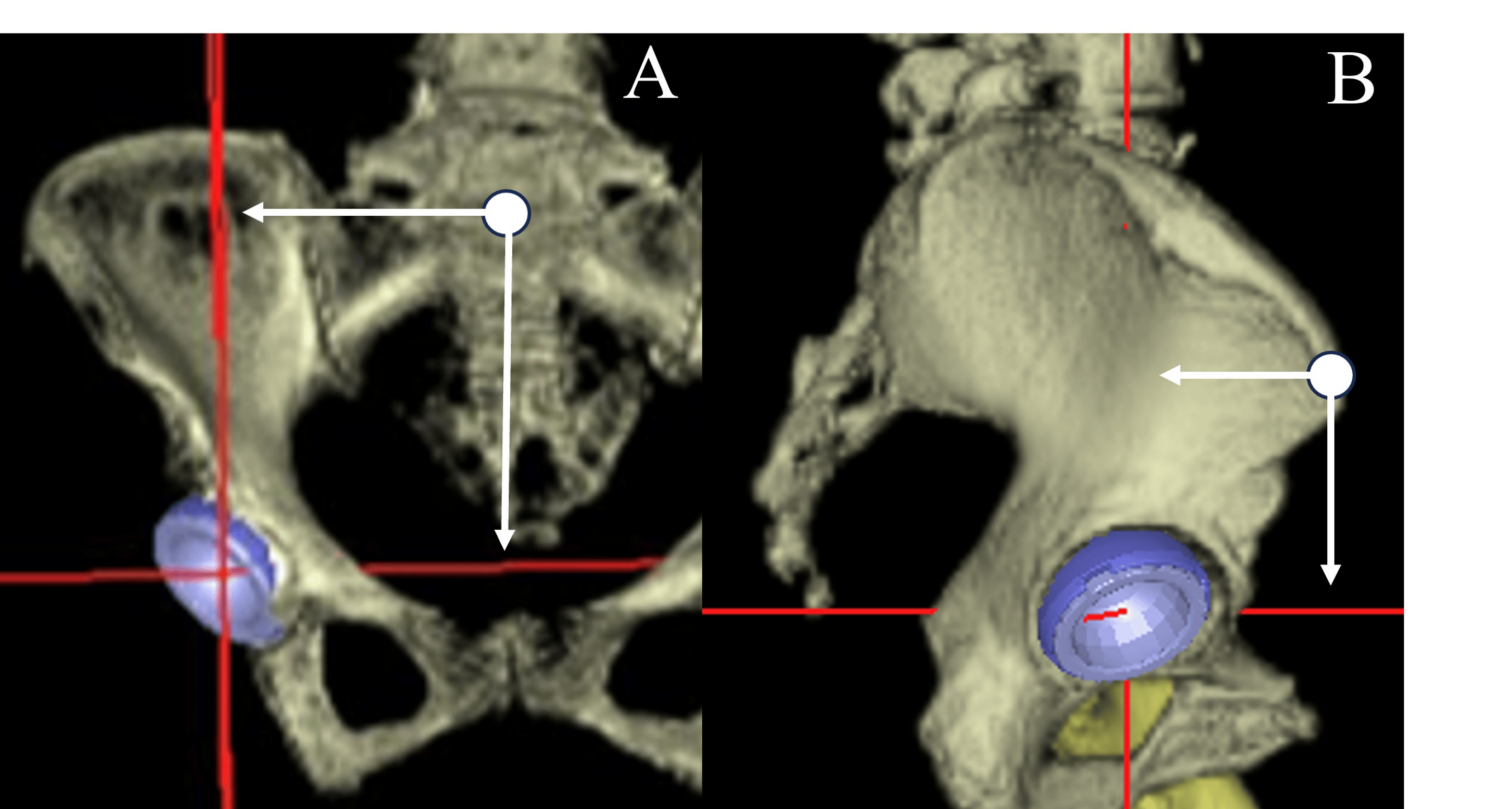
Coordinate system for cup position assessment The cup center coordinates are defined in three-dimensional CT: X (transverse), Y (sagittal), and Z (longitudinal) axes. A: Front view of the pelvis; B: Lateral view of the pelvis The white dots represent the center of both ASISs. The white arrows illustrate the distances from the center of both ASISs to the center of the acetabular cup in the transverse, sagittal, and longitudinal planes, respectively. The three-dimensional coordinates of the center of the acetabular cup are determined based on measured distances. ASIS: Anterior superior iliac spine

The transverse axis, also referred to as the X-axis, was connected to the bilateral ASISs, while the longitudinal axis, or Z-axis, was positioned perpendicularly to the X-axis, running parallel to the FPP. In the sagittal view, the Y-axis was oriented perpendicularly to the Z-axis (Figure 4).

The distance from the center of both ASISs was set as coordinates along the X, Y, and Z axes, and the position of the cup in 3D space was defined based on these coordinates. The software in the navigation system can measure the distance from the center of the line connecting the two ASISs measured on the FPP coordinates to the center of the head on the X, Y and Z axes, and display it as preoperative planning and postoperative head position information (Table 1).

**Table 1:** Location of head center displayed on navigation monitor Distance from the center of the line connecting both ASISs to the center of the head as measured in the FPP coordinates of the navigation software for the same case shown in Figures 1 and 3. ASIS: Anterior superior iliac spine; FPP: Functional pelvic plane

	Transverse (X-axis)	Sagittal (Y-axis)	Longitudinal (Z-axis)
Preoperative planning (mm)	80	60	61
After surgery (mm)	79	60	62

To evaluate the accuracy of the cup position using the navigation system, we calculated the absolute difference between the preoperative plan (Figure 1) and the postoperative cup position (Figure 3) on the three-dimensional template. All patients were classified into four groups according to the Crowe classification system: Crowe I, II, III and those without DDH [22]. Cases were further divided into two groups based on the presence or absence of a double floor, which was determined by preoperative X-ray imaging. We examined whether there was a difference in the absolute discrepancy of the cup position for each group. There were no surgeries for Crowe IV cases during the study period. We also investigated whether there were differences in cup placement accuracy with respect to variations in surgical approach (e.g., modified Watson-Jones approach and Harding approach).

Statistical analysis

Statistical analyses were conducted using IBM SPSS Statistics 30 (IBM Corporation, 2016, USA). The Shapiro-Wilk test was used to assess the normality of all continuous variables. Data that did not follow a normal distribution were analyzed using non-parametric tests. In this study, we analyzed the entire cohort and determined that the effect of individual outliers would be statistically reduced because the sample size was sufficient. Therefore, we did not detect or exclude individual outliers. Quantitative data on the absolute discrepancy in cup placement, comparing cases with or without a double floor, and using two different surgical approaches were all analyzed using the Mann-Whitney U test. Comparisons between the Crowe classification types were conducted using the Kruskal-Wallis test for non-normally distributed data. Intra-observer reliability was assessed using Spearman’s rank correlation coefficient. A result was considered statistically significant if its p value was less than 0.05.

## Results

Patient demographics are presented in Table 2.

**Table 2:** Patient demographics THA: Total hip arthroplasty; OA: Osteoarthritis; ION: Idiopathic osteonecrosis; SIF: Subchondral insufficiency fracture; FNF: Femoral neck fracture; RA: Rheumatoid arthritis; RDC: Rapidly destructive coxarthropathy; DDH: Developmental dysplasia of the hip Data are presented as mean ± SD.

Variable	
Age at THA (years)	68 ± 11
Gender	
Total	160 (173 hips)
Female	130 (81.3%)
Male	30 (18.8%)
Height (cm)	156 ± 8.2
Weight (kg)	59 ± 13
BMI (kg/m²)	24 ± 4.2
Disease	
OA	145 (83.8%)
ION	13 (7.5%)
SIF	4 (2.3%)
FNF	7 (4.0%)
RA	3 (1.7%)
RDC	1 (0.6%)
Approach	
Modified Watson-Jone approach	74 (42.8%)
Modified Hardinge approach	99 (57.2%)
Crowe classification	
DDH (-)	30 (17.3%)
Type I	90 (52.0%)
Type II	37 (21.4%)
Type III/IV	16 (9.2%)
Double floor	
Without	59 (34.1%)
With	114 (65.9%)

The absolute discrepancies between preoperative plans and postoperative measurements were 1.7 (range 0-8) mm on the X-axis, 2.3 (range 0-12) mm on the Y-axis, and 2.2 (range 0-11) mm on the Z-axis for all patients (Table 3).

**Table 3:** Absolute differences between preoperative planning cup positions and postoperative cup position measurements in three directions Data are presented as mean ± SD (range).

	X-axis	Y-axis	Z-axis
Absolute differences (mm)	1.7 ± 1.5 (0-8)	2.3 ± 2.2 (0-12)	2.2 ± 2.1 (0-11)

The average error in the cup placement position was within 2.5 mm in all directions for each Crowe classification. Additionally, no significant differences in error were observed between the Crowe classifications. P-values were 0.202 for the X-axis, 0.919 for the Y-axis, and 0.253 for the Z-axis (Table 4).

**Table 4:** Effects of Crowe classification on the discrepancy of cup position Data are presented as mean ± SD. The Kruskal-Wallis test was used for statistical analysis.

	DDH (-)	Type I	Type II	Type III/IV	P-value
X-axis (mm)	1.5 ± 1.1	1.5 ± 1.5	1.8 ± 1.5	2.3 ± 1.9	0.202
Y-axis (mm)	2.4 ± 2.2	2.2 ± 2.0	2.3 ± 2.4	2.3 ± 1.9	0.919
Z-axis (mm)	2.0 ± 2.1	2.4 ± 2.2	2.1 ± 2.1	1.6 ± 1.4	0.253

The variation in measurements in all three dimensions was not significantly different between groups with or without a double floor. P-values were 0.158 on the X-axis, 0.062 on the Y-axis, and 0.571 on the Z-axis; however, the subgroup with a double floor showed a trend toward greater discrepancy in all directions (Table 5).

**Table 5:** Effects of double floor on discrepancy of cup position Data are presented as mean ± SD. The Mann-Whitney U test was used for statistical analysis.

	Without Double Floor	With Double Floor	P-value
X-axis (mm)	1.4 ± 1.3	1.8 ± 1.6	0.158
Y-axis (mm)	1.9 ± 2.0	2.5 ± 2.2	0.062
Z-axis (mm)	2.0 ± 1.8	2.3 ± 2.2	0.571

In addition, the variation in measurements in all three dimensions was not significantly different between the modified Watson-Jones approach and modified Hardinge approach (Table 6).

**Table 6:** Effects of variability in surgical approaches Data are presented as mean ± SD. The Mann-Whitney U test was used for statistical analysis.

	Modified Watson-Jone Approach	Modified Hardinge Approach	P-value
X-axis (mm)	1.5 ± 1.3	1.8 ± 1.6	0.227
Y-axis (mm)	2.0 ± 1.5	2.5 ± 2.5	0.675
Z-axis (mm)	2.0 ± 1.6	2.3 ± 2.4	0.685

Intra-observer reliability showed a strong positive correlation, with a Spearman’s rank correlation coefficient of 0.732 (95% CI: 0.479-0.870, n = 30; p < 0.001).

## Discussion

In THA for secondary OA due to DDH, placement of the acetabular cup in the true acetabular site is essential for the reconstruction of a normal hip rotation center and long-term survival of the implant [24]. However, in cases of DDH, if a cup is placed in the original acetabulum, contact with the host bone may be insufficient, resulting in poor fixation of the cup. Additionally, a bulk bone graft may be required for fortified weight-bearing, though there remains a debate about the long-term outcomes of bulk bone grafts [25]. Therefore, medialization or high hip center of the cup tends to be acceptable in cases of DDH [14]. Takao et al. proposed the concept of cup CE angle and reported that failure to achieve a cup CE angle of 8.4° or greater leads to poor outcomes [26]. We also refer to this report when preoperatively planning, allowing for medialization or a high hip center so that the cup CE angle is 10° or greater. The accuracy of cup placement was also reported in similar previous studies. Iwana et al. reported that the absolute discrepancy from the preoperative plan is 1.9 mm on the X-axis, 1.4 mm on the Y-axis, and 1.9 mm on the Z-axis [27]. Furthermore, in their report on robotic THA, Okazaki et al. reported good placement accuracy with absolute deviations from the preoperative plan of 1.4 mm on the X-axis, 1.8 mm on the Y-axis, and 1.8 mm on the Z-axis [9]. Okazaki et al. and Tamaki et al. reported that there was no significant difference in the accuracy of cup placement position between robotic THA and navigated THA, while Ando et al. reported that the accuracy of cup placement position of robotic THA was superior to that of navigated THA [9,28,29]. Accuracy of cup placement in robotic THA may be higher than conventional CT-navigated THA.

On the other hand, there have been few reports of studies investigating whether the accuracy of cup placement varies with the degree of DDH. Tsutsui et al. investigated the difference in accuracy according to the Crowe classification in the same way as the present study and reported no difference in accuracy [23]. In addition, Hayashi et al. reported that there was no difference in the accuracy of cup placement between DDH and non-DDH cases in robotic THA [30]. In the present study, there was no difference in the accuracy of cup placement in either the X, Y, or Z-axis directions, even in Crowe II and III cases with severe superior subluxation. On the other hand, there are no reports on the difference in accuracy of cup placement in cases with a double floor due to the lateralization of the femoral head. In the present study, there was no statistically significant difference in accuracy between cases with and without a double floor, but there was a decrease in accuracy in cases with a double floor in all X, Y and Z directions. In cases where a double floor is present, the accuracy of the cup placement position may be reduced. Our hypothesis is that in cases with a double floor, there are those in which the original acetabulum could have been pointed during intraoperative registration, and cases in which the original acetabulum could not have been pointed due to osteophyte formation on the secondary acetabulum, which may have contributed to the difference in accuracy. However, the location and accuracy of the registration point was not investigated in detail in this study. Furthermore, regarding the depth of the cup, in some cases, even if the cup was inserted after reaming to the planned depth, the press fit was poor and it was necessary to ream deeper. This may have been influenced by technical aspects.

To date, CT-based navigation has been an accurate and useful tool for THA. Precise anatomical and implant information can be obtained through pre- and post-operative CT evaluations, especially in patients with DDH who have distorted acetabular anatomy. However, there are several limitations in the present study. First, because this study was a retrospective observational study, no comparison was made with cup placement without CT navigation. To prove the usefulness of this procedure, a comparison with cup placement without the use of CT navigation is necessary. Furthermore, robotic THA has become increasingly popular in recent years [9,23,28-30]. Although there are questions about cost-effectiveness, we believe that a comparison of accuracy with robotic THA, which is considered to be a more accurate surgical aid than CT navigation, will be necessary. Second, in the present study, the mean deviation during intraoperative surface matching was allowed to be within 1 mm. However, the accuracy of intraoperative surface matching and relationship accuracy of surface mating and cup placement were not investigated. Future studies will be required to examine the accuracy of surface matching and the error in cup positioning. In addition, this study examined discrepancies between preoperative planning and the postoperative placement and did not evaluate the extent to which the surgeon actually tolerated discrepancies in cup position from the preoperative plan during surgery. In fact, during surgery, the surgeon prioritized the press fit of the cup and tolerated a discrepancy from the preoperative plan of up to 2° in angle and 2 mm in distance. Third, the clinical significance cannot be proven because no comparison with clinical scores or long-term results has been conducted.

## Conclusions

The accuracy of the cup positioning using CT navigation was satisfactory with the absolute discrepancy from the preoperative plan in primary THA being 1.7 (range 0 to 8) mm on the transverse axis, 2.3 (range 0 to 12) mm on the sagittal axis, and 2.2 (range 0 to 11) mm on the longitudinal axis. Furthermore, there was no difference in the accuracy of the cup placement with respect to the degree of acetabular deformation according to the Crowe classification or the degree of subluxation of the head with or without a double floor.
